# Circulating microRNA-21 is an early predictor of ROS-mediated damage in subjects with high risk of developing diabetes and in drug-naïve T2D

**DOI:** 10.1186/s12933-019-0824-2

**Published:** 2019-02-25

**Authors:** Lucia La Sala, Simona Mrakic-Sposta, Elena Tagliabue, Francesco Prattichizzo, Stefano Micheloni, Elena Sangalli, Claudia Specchia, Anna Chiara Uccellatore, Silvia Lupini, Gaia Spinetti, Paola de Candia, Antonio Ceriello

**Affiliations:** 1 Department of Cardiovascular and Dysmetabolic Diseases, IRCCS MultiMedica, Via Fantoli 16/15, 20138 Milan, Italy; 2 Institute of Molecular Bioimaging and Physiology, National Research Council, Segrate, Italy; 3 Biostatistic Unit, IRCCS MultiMedica, Milan, Italy; 4 Department of Translational Biomedicine, University of Brescia, Brescia, Italy; 5 University of Milan, Milan, Italy; 6 Institut d’Investigacions Biomèdiques August Pi i Sunyer (IDIBAPS) and Centro de Investigación Biomédica en Red de Diabetes y Enfermedades Metabólicas Asociadas (CIBERDEM), Barcelona, Spain

**Keywords:** miR-21, IGT, T2D, ROS homeostasis, SOD2, Antioxidant response, Prediabetes, Diabetes

## Abstract

**Background:**

Impaired glucose tolerance (IGT) is a risk factor for the development of diabetes and related complications that ensue. Early identification of at-risk individuals might be beneficial to reduce or delay the progression of diabetes and its related complications. Recently, microRNAs emerged as potential biomarkers of diseases. The aim of the present study was to evaluate microRNA-21 as a potential biomarker for the risk of developing diabetes in adults with IGT and to investigate its downstream effects as the generation of reactive oxygen species (ROS), the induction of manganese-superoxide dismutase-2 (SOD2), and the circulating levels of 4-HNE (4-hydroxynonenal).

**Methods:**

To evaluate the prognostic and predictive values of plasmatic microRNA-21 in identifying metabolic derangements, we tested a selected cohort (n = 115) of subjects enrolled in the DIAPASON Study, whom were selected on ADA criteria for 2hPG. Statistical analysis was performed using ANOVA or the Kruskal–Wallis test as appropriate. ROC curves were drawn for diagnostic accuracy of the tests; positive and negative predictive values were performed, and Youden’s index was used to seek the cut-off optimum truncation point. ROS, SOD2 and 4-HNE were also evaluated.

**Results:**

We observed significant upregulation of microRNA-21 in IGT and in T2D subjects, and microRNA-21 was positively correlated with glycaemic parameters. Diagnostic performance of microRNA-21 was high and accurate. We detected significant overproduction of ROS by electron paramagnetic resonance (EPR), significant accumulation of the lipid peroxidation marker 4-HNE, and defective SOD2 antioxidant response in IGT and newly diagnosed, drug-naïve T2D subjects. In addition, ROC curves demonstrated the diagnostic accuracy of markers used.

**Conclusions:**

our data demonstrate that microRNA-21 is associated with prediabetic status and exhibits predictive value for early detection of glucose imbalances. These data could provide novel clues for miR-based biomarkers to evaluate diabetes.

**Electronic supplementary material:**

The online version of this article (10.1186/s12933-019-0824-2) contains supplementary material, which is available to authorized users.

## Introduction

Prediabetes (also known as impaired glucose tolerance, IGT) is an asymptomatic and heterogeneous condition characterized by a transitional state of hyperglycaemia that is not high enough to be diagnosed as diabetes but appears before the disease onset. Emerging data indicate that IGT is marked by metabolic abnormalities typical of type 2 diabetes (T2D), such as insulin resistance (IR), obesity and the development of cardiovascular disorders, whose central trigger factors have been recognized as high and prolonged oxidative stress (Ox-S) within tissues [1, 2]. As prediabetes is a strong predictor for cardiovascular disease [3], its typical metabolic derangements may expose people to a higher risk of developing T2D, and it may have severe consequential complications; therefore, prediabetics are a target population for lifestyle intervention. However, many individuals with prediabetes progress to T2D despite the considerable benefits of lifestyle modification. Therefore, early identification of populations at high risk for diabetes would improve prevention strategies, avoid treatment burden for more low-risk individuals, and greatly improve patient care.

The use of biomarkers is increasing, and technical advancements allow more accurate identification of processes involved in pathological progression. Glucose intolerance is generally established after a 75-g glucose load test; IGT is defined as a 2-h plasma glucose (2hPG) level of 140 mg/dL ≤ 2hPG < 200 mg/dL (or 7.7 mmol/L ≤ 2hPG < 11.1 mmol/L); and T2D is defined as a 2hPG level ≥ 200 mg/dL [4]. Numerous studies have confirmed that 2hPG values are a more accurate diagnostic measure for diabetes than when using only fasting plasma glucose (FPG) or haemoglobin A1c (HbA1c) [5, 6]. However, to implement prevention strategies, it is crucial to identify these subjects before the appearance of clinical symptoms. Recently, alternative biomarkers such as circulating microRNAs (miRNAs) have been shown to be associated with T2D [7, 8] and are also reliable markers for other diseases [9]. MiRNAs are small, non-coding endogenous RNAs, with 18–22 nucleotides in length, able to modulate the expression of complementary messenger RNAs by pairing to the untranslated region (3′-UTR) [10] and involved in regulating most molecular processes. There is evidence that miRNAs are linked to metabolic processes, including insulin metabolism and glucose homeostasis [11]. Interestingly, miRNAs alter gene expression of insulin-producing and insulin-sensitive tissues; examples include the pancreas regulated by miR-375 [12], the liver by miR-122 [13], and vasculature. Circulating miRNAs have been detected in the blood stream, where they exhibit high stability and reproducibility. Circulating miRNAs are well suited to provide associated clinical information about pathological-physiological conditions, suggesting their important role in pathogenesis, early diagnosis, and outcomes of diabetes. Although the precise mechanisms of miRNA release into the blood stream are only partially understood, it seems that miRNAs reach the circulatory system through a complex release mechanism from cells by strict association with extracellular vesicles (EVs) or carrier proteins [14].

Recent evidences reported circulating miRNAs are candidate as new biomarkers of IR and adiposity [15], also for monitoring the response to therapy with respect to glycaemic target [16, 17], and for diabetes complications [18, 19], but their identification in pre-diabetes is still under evaluation. Parrizas et al. [20] found that two candidate miRNAs, namely miR-192 and miR-193b, are markers of pre-diabetes in a Spanish cohort; de Candia et al. [21] showed a unique miRNA signature related to prediabetics with particular regards to disease progression; Yan et al. [22] screened and validated differentially expressed plasma miRNAs in pre-diabetes and newly diagnosed T2D. Although these microRNAs may identify pre-diabetes, a large-scale validation program is lacking.

In this work, we hypothesised that miR-21 could be an excellent candidate to monitor hyperglycaemic injury in plasma; this is supported by its role in ROS homeostasis [2, 23] and by our observation of impaired expression of manganese-superoxide dismutase 2 (SOD2) in a cellular model of glucose variability (GV) [24], which suggested the damaging effects of hyperglycaemia on the antioxidant defence system, via KRIT1-modulated miR-21 expression. We aimed to develop a miRNA-based method that, when coupled with canonical markers used for diagnosis and prognosis of prediabetes (or diabetes), would be helpful in detection of glycaemic status to understand the molecular mechanisms of glucose abnormalities progression.

## Materials and methods

### Participants and Setting

Participants were recruited within DIAPASON (diabetes prediction and screening observational) Study cohort (n = 115), a diabetes prevention programme conducted in Milan (Italy). Participants were selected by general practitioners using the Finnish Diabetes Risk Score (FINDRISC) questionnaire, and a score of ≥ 9 (based on IGLOO study results in detecting individuals with glucose abnormalities) [25] indicated eligibility. Participants signed informed consent prior to laboratory screening. Since 2-h plasma glucose (2hPG) values are more accurate diagnostic measures for T2D and IGT than are only FPG or HbA1c [5, 6], we used the American Diabetes Association (ADA) criteria to identify normo-glucose tolerance (NGT), impaired glucose tolerance (IGT) and newly diagnosed, drug-naïve type 2 diabetes subjects (T2D); we used 2hPG values in the 75-g oral glucose tolerance test (OGTT): NGT < 140 mg/dL (< 7.8 mmol/L); IGT, between 140 and 199 mg/dL (7.8 mmol/L and 11.0 mmol/L); and T2D, ≥ 200 mg/dL (≥ 11.1 mmol/L) [4]. The DIAPASON protocol was approved by the institutional review boards of the IRCCS MultiMedica [protocol number 24/2012(153)].

### Plasma separation and laboratory testing

Approximately 5 mL of venous blood sample was extracted in an ethylenediaminetetraacetic acid (EDTA) anticoagulant tube at room temperature. The venous blood sample was centrifuged at 3000 r/min for 10 min. The level of haemolysis in plasma samples was assessed by spectrophotometry as ratio between the optical density of 414 and 375 nm [26, 27] (see Additional file 1). Fasting plasma glucose (FPG) was detected by the Slein method using a Siemens analyser (Germany); triacylglycerol (TAG) and total cholesterol (TC) were measured by an automated enzymatic colorimetric test (Siemens); glycosylated haemoglobin (HbA1c) was detected by an automated analyser (Tosoh, Japan); and insulinaemia levels were detected by a CentaurusXP automatic biochemical analyser (Siemens). The homeostasis model assessment for insulin resistance (HOMA-IR) was consequently calculated [fasting plasma glucose (mg/dL) × fasting insulin (uU/mL)/405]. Microalbuminuria (m-ALB) was detected by kinetic nephelometry using IMMAGE (Beckman Coulter, Inc.) in urine samples previously centrifuged for 10 min at 3000×*g* to avoid cellular debris. All procedures were in strict accordance with the kit instructions. We used the OGTT to perform the 2-h and 1-h oral glucose tolerance tests.

### RNA extraction and miRNA determination with real-time PCR analysis

Total RNA was extracted from 100 μL of plasma from subjects using an RNA purification kit (NorgenBiotek, Thorold, ON, Canada) following the manufacturer’s instructions. Plasma was therefore centrifuged at 13,000*g* for 5 min at 4 °C in order to avoid platelets interferences.

Before RNA extraction, 5 μL of *cel*-miR-39 [(synthetic *Caenorhabditis elegans*-miR-39), purchased by Applied Biosystems, Life Technologies, Grand Island, NY, USA] was spiked into plasma to ensure efficiency RNA recovery. The TaqMan MicroRNA Reverse Transcription Kit (Thermo-Fisher) was used to reverse-transcribe miRNAs as recommended by the manufacturer, and endogenous levels of miR-21 were measured in plasma. Real-time qPCR was performed with a QuantStudio 6 flex (Applied Biosystems, Foster City, CA, USA) detection system. Data were obtained as Ct values, and the 2^−ΔCt^ method was used in the analysis. For analysis of miRNA expression levels, external normalization to *cel*-miR-39 was applied.

### Determination of ROS by electron paramagnetic resonance (EPR)

ROS generation was detected in sera of NGT, IGT and drug-naïve T2D subjects by EPR spectroscopy (EPR spectrometer, Bruker, Karlsruhe, Germany) using the EPR method [28]. Sera were incubated with 1 mM CMH (1-hydroxy-3-methoxycarbonyl-2,2,5,5-tetramethylpyrroline) probe prepared in buffer (Krebs-Hepes buffer (KHB) containing 25 μM deferroxamine methane-sulfonate salt (DF) chelating agent and 5 μM sodium diethyldiothio-carbamate trihydrate (DETC) at pH 7.4). Spectra were recorded and analysed by standard software (Win EPR 2.11, Bruker).

### Determinations of plasmatic 4-hydroxynonenal (HNE) and human SOD2

One hundred μL of plasma samples in duplicate were used to analyse HNE-protein adduct concentrations with a commercially available immunoassay kit following the manufacturer’s instructions (Oxiselect, Cell Labs, San Diego, CA, USA). One hundred μL of 20-fold diluted plasma for SOD2 (Abnova, UK) was used according to the manufacturer’s instructions.

### Statistical analysis

The χ^2^ test was used to compare sex frequencies among diagnostic groups; continuous variables were compared among groups by the F-test (normally distributed variables) or the Kruskal–Wallis test (non-normally distributed variables). Pairwise comparisons were also evaluated. Correlations between miR-21, HNE, SOD2 and ROS levels and clinical parameters were assessed by Spearman correlation coefficients. Receiver operating characteristic (ROC) curves were drawn for miR-21, HNE, SOD2 and ROS levels, and Youden’s Index was used to identify their best cut-offs to discriminate between groups. Sensitivity (SE), specificity (SP), positive and negative predicted values (PPV and NPV) and positive and negative likelihood ratios (LR+ and LR−) were also calculated. Associations between miR-21, HNE, SOD2, ROS and diagnostic groups were evaluated by multivariate unconditional logistic regression models comparing IGT (and T2D) with NGT after adjusting for significant variables by univariate analysis. In addition, stepwise regression analysis was performed to identify the best regression models. Odds ratios (ORs) and 95% confidence intervals (CIs) were calculated. ROC curves were drawn for all models, and areas under the ROC curve (AUCs) were compared using a nonparametric approach [29]. To compare the performance of miR-21, Hb1Ac and FPG in predicting IGT (and T2D) compared with NGT, we drew ROC curves and then compared AUCs. All reported p-values were two-sided, and p-values < 0.05 were considered statistically significant. Statistical analyses were performed with SAS Software 9.4.

## Results

### Circulating miR-21 identify the hyperglycaemic state in high-risk subjects

**Fig. 1:**
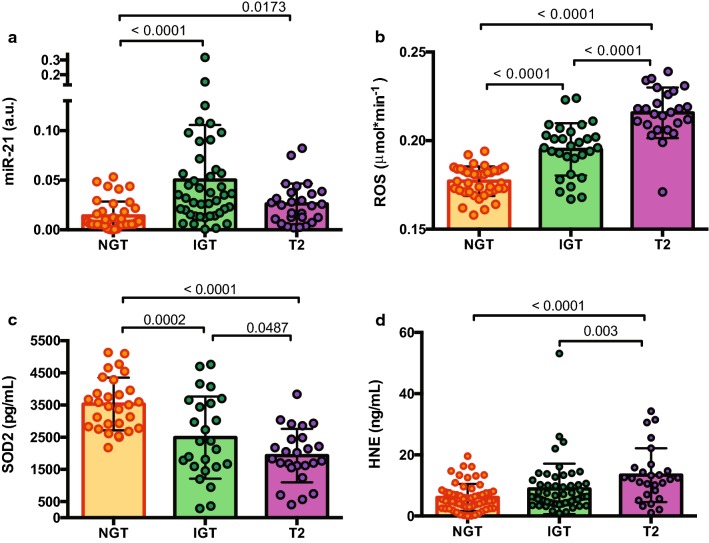
Identification of impaired glucose tolerance (IGT) and T2D drug-naïve phenotypes by elevated c-miR-21 expression in plasma samples. **a** Scatter dot plot of miR-21 expression in IGT and T2D in which miR-21 was normalised to cel-miR-39 expression using the comparative Ct method in a high-risk cohort (N = 109) screened on 2hPG values: NGT, n = 39; IGT, n = 43 and T2D n = 27. Data are presented as the mean (± SD). ANOVA p < 0.0001. **b** Scatter dot plot of ROS extracellular release (measured by EPR instruments) in sera showing significant increases in IGT and T2D subjects (p < 0.0001 for both), demonstrating the potential role for c-miR-21 in oxidative stress. One-way ANOVA followed by Tukey’s post hoc test. **c** Plasma concentration SOD2 (pg/mL) in controls (n = 25), IGT (n = 25) and T2 diabetes (n = 24) subjects. **d** Plasma concentration of HNE (μg/mL) in controls (n = 37), IGT (n = 39) and T2 diabetes subjects (n = 27). Values are mean (± SD) of the concentration of HNE

We evaluated the potential role of circulating miR-21 in identifying glycaemic status, defined as prediabetes (IGT) and newly diagnosed, drug-naïve T2D on the basis of ADA criteria for 2hPG, in a selected cohort from DIAPASON (n = 109). qPCR was used for miR-21 detection in plasma of normoglycaemic subjects (NGT, n = 39), prediabetics (IGT, n = 43), and subjects with newly diagnosed, drug-naïve T2D (T2D, n = 27). Our data showed that the mean value of miR-21 plasma level was significantly higher in IGT and T2D compared to NGT (p < 0.0001 and p = 0.0173, respectively) (Table 1; Fig. 1a), suggesting a strict relationship between miR-21 and different glycaemic statuses. Interestingly, the correlation analysis revealed a significant positive association of circulating miR-21 with clinically relevant glycaemic parameters, such as 1-h plasma glucose (1hPG) and 2hPG with insulinaemia, HOMA-IR, and ROS generation (Spearman *ρ* = 0.2, p = 0.008), thereby corroborating the link to oxidative stress (Table 2). In addition, circulating miR-21 was negatively correlated with plasmatic SOD2 protein levels (Spearman *ρ* = − 0.34, p = 0.002).

**Table 1:** Characteristic baseline of subjects

	NGT		IGT			T2			Overall
	*n*	%male	*n*	%male	p (vs NG)	*n*	%male	p (vs NG)	p*
*Sex*	*44*		*44*		0.085	*27*		0.8	0.19
F	*29*	65.91	*21*	47.73		*17*	62.96		
M	*15*	34.09	*23*	52.27		*10*	37.04		

	NGT		IGT			T2			Overall
	*n*	Mean (± SD)	*n*	Mean (± SD)	p (vs NG)	*n*	Mean (± SD)	p (vs NG)	p*
Age (years)	*44*	59.3 (9.82)	*44*	61.52 (12.7)	0.2743	*27*	61.69 (7.59)	0.6886	0.283
BMI (kg/m^2^)	*44*	25.11 (3.32)	*44*	27.02 (3.61)	***0.029***	*27*	29.26 (5.83)	***0.0022***	***0.001***
FPG (mg/dL)	*44*	86.61 (15.23)	*44*	96.36 (12.01)	**<** ***0.0001***	*27*	110.56 (14.55)	**<** ***0.0001***	**<** ***0.0001***
1hPG (mg/dL)	*41*	127.93 (33.24)	*44*	189.86 (34.56)	**<** ***0.0001***	*27*	233.15 (27.42)	**<** ***0.0001***	**<** ***0.0001***^**#**^
2hPG (mg/dL)	*44*	100.89 (22.34)	*44*	161.36 (17.63)	**<** ***0.0001***	*27*	238.78 (39.21)	**<** ***0.0001***	**<** ***0.0001***
HbA1c (%)	*44*	5.8 (0.38)	*44*	6.2 (0.41)	**<** ***0.0001***	*27*	6.64 (0.6)	**<** ***0.0001***	**<** ***0.0001***
INS (mIU/L)	*42*	16.61 (21.05)	*44*	18.27 (23.1)	***0.0183***	*25*	21.04 (20.18)	***0.0049***	***0.0021***
HOMA-IR	*42*	3.43 (4.2)	*43*	4.48 (5.79)	***0.006***	*25*	5.9 (5.94)	***0.0001***	**<** ***0.0001***
TC (mg/dL)	*44*	204.45 (34.68)	*44*	206.14 (35.43)	0.8196	*27*	200.41 (32.6)	0.6323	0.7922^#^
HDL (mg/dL)	*44*	57.52 (12.51)	*44*	51.8 (12.82)	***0.0369***	*27*	51.96 (12.89)	0.0765	0.0719^#^
LDL (mg/dL)	*44*	125.68 (27.84)	*44*	130.28 (30.66)	0.457	*27*	123.13 (27.62)	0.7187	0.5674^#^
TAG (mg/dL)	*44*	106.27 (55.95)	*44*	120.25 (49.68)	0.1414	*27*	126.59 (62.9)	0.1963	0.0995
m-ALB (mg/dL)	*42*	7.84 (10.36)	*38*	26.49 (67.67)	***0.0117***	*25*	18.19 (32.1)	***0.0252***	***0.005***
miR-21 (a.u.)	*39*	0.014 (0.01)	*43*	0.05 (0.056)	**<** ***0.0001***	*27*	0.026 (0.021)	***0.0173***	**<** ***0.0001***
ROS (μmol/min)	*40*	0.18 (0.01)	*29*	0.2 (0.01)	**<** ***0.0001***	*25*	0.22 (0.01)	**<** ***0.0001***	**<** ***0.0001***
SOD2 (pg/mL)	*29*	3532 (818.6)	*25*	2489.7 (1274.9)	***0.0002***	*25*	1929.4 (830.7)	**<** ***0.0001***	**<** ***0.0001***^**#**^
HNE (μg/mL)	*37*	4.94 (3.66)	*39*	7.83 (8.6)	0.1309	*27*	13.37 (8.78)	**<** ***0.0001***	**<** ***0.0001***

The italic and bolditalic values are statistical significance

BMI, body mass index; FPG, fasting plasma glucose; 1hPG, 1-hour plasma glucose; 2hPG, 2-hour plasma glucose; HbA1c, glycated hemoglobin A1c; INS, insulinemia; HOMA-IR, homeostatic model assessment for insulin resistance; TC, total cholesterol; HDL, high-density lipoprotein; LDL, low density lipoprotein; TAG, triacylglycerol; m-ALB, microalbuminuria; miR-21, microRNA-21; HNE, hydroxynonenal; SOD2, superoxide dismutase 2; ROS, reactive oxygen species

* Kruskal–Wallis test; ^#^F test

**Table 2:** Spearman correlation matrix for variables

	miR-21		HNE		SOD2		ROS	
	*r*	p*	*r*	p*	*r*	p*	*r*	p*
Age (years)	0.01	0.9	− 0.009	0.9	− 0.16	0.17	0.03	0.8
BMI (kg/m^2^)	0.1	0.25	− 0.008	0.9	− ***0.3***	***0.008***	0.24	0.02
Fasting PG (mg/dL)	0.16	*0.08*	***0.35***	***0.0003***	− ***0.4***	***0.007***	***0.5***	***<*** ***0.0001***
1hPG (mg/dL)	***0.2***	***0.008***	***0.35***	***0.0003***	− ***0.4***	***0.007***	***0.6***	***<*** ***0.0001***
2hPG (mg/dL)	***0.3***	***0.005***	***0.45***	***<*** ***0.0001***	− ***0.5***	***<*** ***0.0001***	***0.7***	***<*** ***0.0001***
HbA1c (%)	0.1	0.2	**0.29**	**0.003**	− ***0.4***	***0.0003***	***0.5***	**<** **0.0001**
Insulinemia (mIU/L)	***0.22***	***0.02***	0.06	0.5	− ***0.3***	***0.0097***	0.2	*0.055*
HOMA-IR	***0.24***	***0.01***	0.13	0.2	− ***0.3***	***0.003***	***0.3***	***0.004***
Cholesterol (mg/dL)	− 0.08	0.4	− 0.09	0.34	0.1	0.4	0.11	0.3
HDL (mg/dL)	− 0.1	0.3	0.11	0.24	***0.3***	***0.003***	− 0.1	0.33
LDL (mg/dL)	− 0.06	0.5	− 0.05	0.64	0.015	0.9	0.04	0.65
TAG (mg/dL)	0.05	0.5	− ***0.27***	***0.004***	− 0.15	0.2	0.2	*0.05*
m-ALB	− 0.004	0.96	0.05	0.6	− 0.02	0.9	***0.3***	***0.0025***
ROS (μmol/min)	***0.2***	***0.0084***	***0.33***	***0.002***	− ***0.4***	***0.0006***	–	–
SOD2 (pg/mL)	− ***0.34***	***0.002***	− 0.2	*0.08*	–	–	− ***0.4***	***0.0006***
HNE (μg/mL)	0.06	0.55	–	–	− 0.2	*0.08*	***0.33***	***0.002***

In bold italics, statistical significance

### IGT and T2D phenotypes exhibit increased oxidative stress and defective antioxidant response

Plasma has a biological role in counteracting toxic compounds due to the presence of antioxidant substances. Although it is unclear whether plasma is the main site of ROS production, values of ROS were detected by EPR. We measured ROS absolute concentrations in NGT (n = 40), IGT (n = 29) and T2D (n = 25) (Fig. 1b). IGT and T2D subjects had increased ROS levels with significant differences compared to NGT (p < 0.0001 for both) (Table 1; Fig. 1b). Interestingly, ROS levels increased more in T2D than in IGT (p < 0.0001). ROS is strongly correlated with all glycaemic parameters, in particular with post-prandial glucose levels such as 1hPG and 2hPG (*ρ* = 0.6 and *ρ* = 0.7, respectively) as well as HOMA-IR (Table 2). Concomitantly, the quantitative determination of protein levels of human SOD2, which has antioxidant activity, revealed a progressive reduction in IGT and T2D (p = 0.0002 and p < 0.0001 compared to NGT, respectively) (Table 1; Fig. 1c). In addition, SOD2 is significantly and negatively associated with all glycaemic parameters, ROS production and insulinaemia and HOMA-IR (Table 2).

### Lipid peroxidation characterizes drug-naïve, newly diagnosed diabetes but not prediabetes

To investigate the free radical-induced lipid damage occurring in plasma and the relative effectiveness of SOD2 plasma antioxidant power in preventing this damage, we assessed the presence of (HNE)-protein adducts in NGT (n = 37), IGT (n = 39) and T2D subjects (n = 27), as these are specific end products of peroxidized lipid. We found a trend towards increased levels of HNE with a significant overall difference (p < 0.0001), although the difference between levels of oxidized protein did not reach statistical significance in NGT vs IGT. However, the levels were significantly higher when we compared T2D with NGT (p < 0.0001), unveiling a status of ROS-damage in diabetes pathogenesis (Table 1; Fig. 1d). Spearman correlation analysis was significant when comparing all glycaemic parameters and ROS production (Table 2).

### Diagnostic specificity and sensitivity of miR-21 for IGT and diabetic subjects

**Fig. 2:**
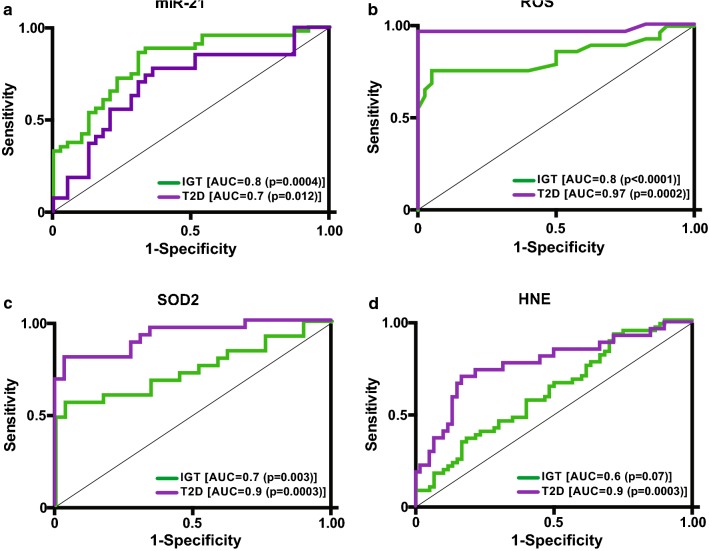
Receiver operator characteristic (ROC) curves generated for sensitivity analysis show diagnostic performances of circulating miR-21 (**a**), ROS (**b**), SOD2 (**c**) and HNE (**d**) in IGT vs NGT (green line) as well as in T2D vs NGT (purple line). The area under the curve (AUC) is reported as the performance measure

**Fig. 3:**
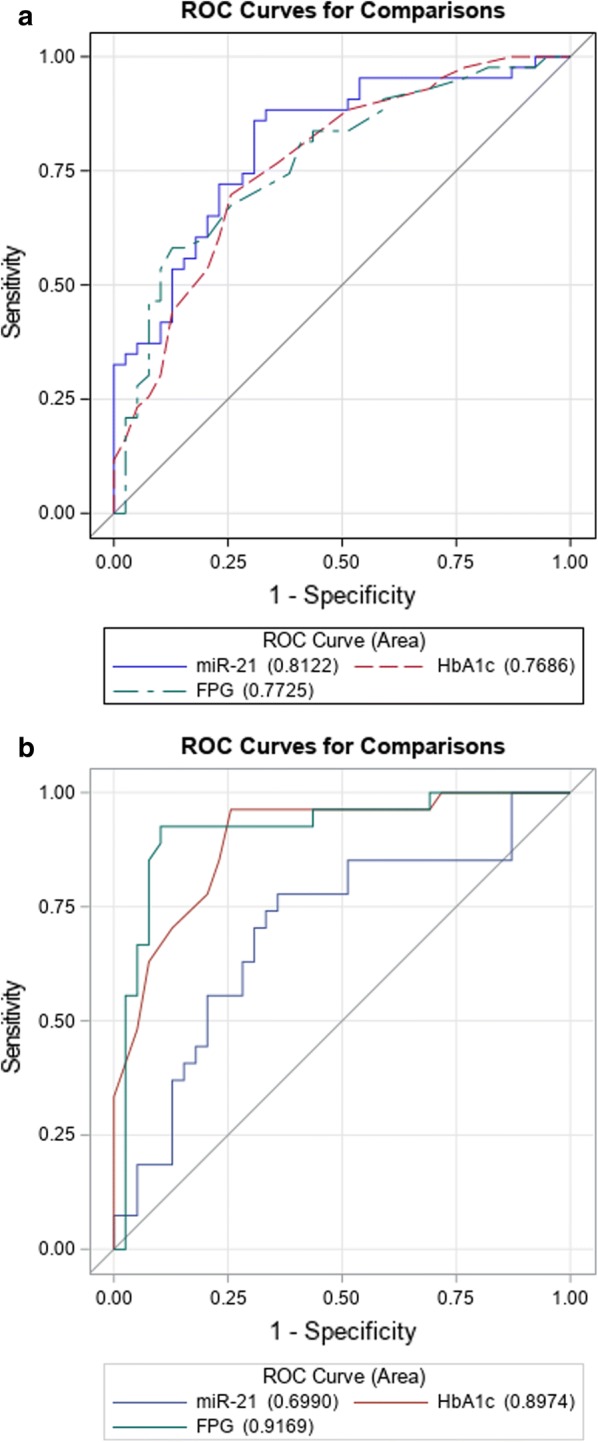
Receiver operator characteristic (ROC) curves generated for sensitivity analysis show diagnostic performances among circulating miR-21, FPG and HbA1c in the diagnosis of IGT (**a**) according to criterion of 2hPG between 140 and 199 mg/dL, and in the diagnosis of T2D (**b**) according to diabetes criterion of 2hPG ≥ 200 mg/dL. The area under the curves (AUC) is reported as the performance measure

To study the diagnostic accuracy of circulating miR-21 as a surrogate biomarker for the IGT or T2D state, a receiving operator characteristic (ROC) curve was drawn. The data demonstrated the diagnostic accuracy of miR-21 (NGT vs IGT, AUC = 0.8; p = 0.0004; NGT vs T2D, AUC = 0.7; p = 0.012) as biomarker of IGT (Fig. 2a; Table 3). Importantly, AUC of miR-21 exhibited higher values in discriminating IGT from NGT; however, when we compared performances between HbA1c and FPG in identifying IGT, miR-21 exhibited better AUC values with respect to canonical markers of glycaemic state (Fig. 3a, b), although it was not significant. The best cut-off for miR-21 in detecting T2D was < 0.0466; this cut-off value achieved a maximum sensitivity of 93% and specificity of 35%. For detecting IGT, ≥ 0.0131 was identified as best cut-off with a maximum sensitivity of 86% and specificity of 69% (Table 3). We also assessed the diagnostic performance, specificity and sensitivity of ROS, SOD2 and HNE in discriminating the IGT and T2D phenotypes (Fig. 2b–d, Table 3); this analysis showed a better AUC in identifying T2D than NGT (Table 3).

**Table 3:** Determination of cut-off optimum truncation point into the discrimination of glycemic status

	Cut-off	SE (%)	SP (%)	PPV (%)	NPV (%)	LR+	LR−	*p* value	AUC
*NGT vs IGT*									
miR-21	≥ 0.0131	86	69	76	82	2.8	0.2	*0.0004*	0.812
SOD2	< 2388.6	56	97	93	72	16.24	0.5	*0.0028*	0.743
HNE	≥ 3.184	92	35	60	81	1.42	0.22	*0.0736*	0.629
ROS	≥ 0.19	76	95	92	84	15.17	0.25	*<* *0.0001*	0.835
*IGT vs T2D*									
miR-21	< 0.0466	93	35	47	88	1.42	0.21	*0.0353*	0.645
SOD2	< 2932.5	92	40	61	83	1.53	0.2	0.0783	0.613
HNE	≥ 10.12	70	82	73	80	3.92	0.36	*0.0275*	0.738
ROS	≥ 0.203	92	72	74	91	3.34	0.11	*0.0005*	0.872

The italic values are statistical significance

SE, sensitivity; SP, specificity; PPV, positive predictive value; NPV, negative predictive value; LR, likelihood ratio; AUC, area under the curve

### miR-21 Logistic regression models to discriminate IGT and NGT subjects

**Fig. 4:**
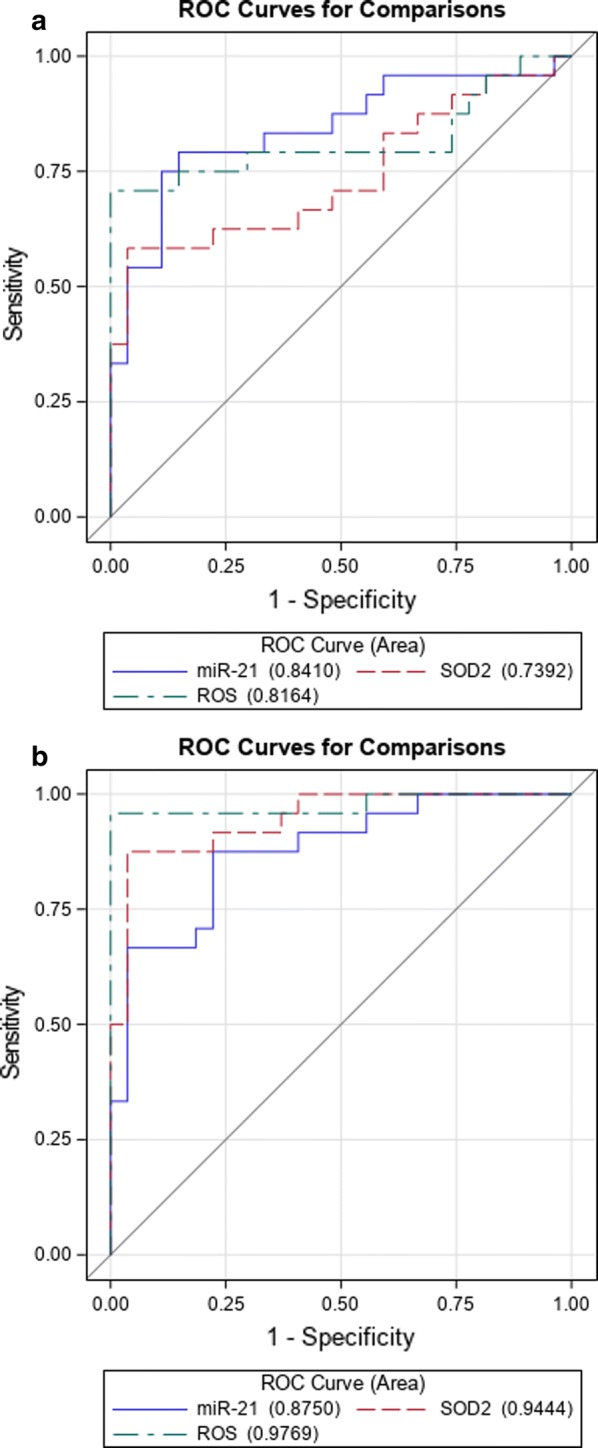
Receiver operator characteristic (ROC) curve for comparison between circulating miR-21, ROS, SOD2 and HNE calculated by multivariable logistic model in prediabetes (**a**) and T2D (**b**)

Table 4 summarizes multivariable models evaluating the association between IGT and biochemical parameters and plasmatic biomarkers, adjusted by sex and body mass index (BMI). Plasma levels of miR-21 were significantly associated with IGT status, with an increasing risk of prediabetes of approximately 6% for each 0.001 unit increase of miR-21 (AUC = 0.835, p = 0.0004) and a 5% increase for diabetes (p = 0.008). ROS was strongly associated with prediabetes [OR (95% CI) = 3.71 (1.93–7.11); p < 0.0001] and with diabetes [OR (95% CI) = 12.06 (2.78–52.3); p = 0.0009]. Increased SOD2 reduced the risk of prediabetes [OR (95% CI) = 0.92 (0.86-0.98); p = 0.0066] and diabetes [OR (95% CI) = 0.7 (0.57–0.87); p = 0.0009]. Figure 4a shows the comparison between ROC curves of logistic models, adjusted for age and BMI; the model with miR-21 has the best performance in identifying IGT vs NGT (AUC = 0.84) compared to ROS and SOD2 (Fig. 4a), but the differences are not statistically significant. Otherwise, in discrimination of T2D vs NGT, ROS had the best performance (Fig. 4b) with respect to miR-21 and SOD2, but no significant difference was observed among the values.

**Table 4:** Discrimination based on multivariable logistic regression models

	OR (95% CI)	*p*	OR (95% CI)	*p*	OR (95% CI)	*p*	OR (95% CI)	*p*
*IGT vs NGT*								
Sex F	0.39 (0.13–1.18)	0.0963	0.57 (0.21–1.50)	0.2525	0.72 (0.21–2.49)	0.6027	0.56 (0.15–2.13)	0.3925
BMI	1.17 (0.99–1.39)	0.0582	*1.20 (1.02*–*1.41)*	*0.0331*	1.09 (0.91–1.30)	0.3644	1.14 (0.94–1.38)	0.181
miR-21	*1.06 (1.03*–*1.10)*	*0.0004*						
HNE			1.01 (0.99–1.03)	0.0638				
SOD2					*0.92 (0.86*–*0.98)*	*0.0066*		
ROS							*3.71 (1.93*–*7.11)*	*<* *0.0001*
AUC	0.835		0.717		0.754		0.847	
*T2D vs NGT*								
Sex F	0.61 (0.18–2.05)	0.4241	0.77 (0.17–3.60)	0.7394	0.78 (0.11–5.56)	0.8007	4.00 (0.06–248.71)	0.5108
BMI	*1.34 (1.12*–*1.59)*	*0.0012*	*1.49 (1.16*–*1.91)*	*0.002*	*1.28 (1.06*–*1.55)*	*0.0115*	1.32 (0.89–1.95)	0.1627
Mir21	*1.05 (1.01*–*1.09)*	*0.0077*						
HNE			*1.04 (1.02*–*1.06)*	*0.0001*				
SOD2					*0.70 (0.57*–*0.87)*	*0.0009*		
ROS							*12.06 (2.78*–*52.30)*	*0.0009*
AUC	0.812		0.925		0.948		0.977	

The italic values are of statistical significance

Logistic models: ORs/miR-21 are calculated for 0.001 unit increase; ORs/HNE are calculated for 0.1 unit increase; ORs/SOD2 are calculated for 100 unit increase; ORs/ROS are calculated for 0.01 unit increase

### miR-21, SOD2 and ROS as the best model to identify IGT

Since the increase of miR-21 is not harmonious during the different status of IGT and T2D [(although in the model adjusted for ROS (data not shown) is leading to miR-21 OR (95% CI) = 1.07 (1.02–1.13), p = 0.0064)] such as those of ROS and inversely for SOD2 (Fig. 1b, c), we sought to identify the best multivariable logistic model for IGT by performing a stepwise logistic regression. We evaluated sex, BMI, miR-21, ROS, SOD2 and HNE. The best model was composed of miR-21, SOD2 and ROS with AUC reaching an accuracy of 94% (Table 5) for identification of IGT.

**Table 5:** Stepwise regression model

	OR (95% CI)	*p*
miR-21	*1.09 (1.01–1.17)*	***0.0266***
SOD2	*0.85 (0.73–0.99)*	***0.0358***
ROS	*7.34 (1.59–33.9)*	***0.0106***
AUC	0.944	

The bolditalic values are statistical significance. The italic is the value of OR that was resulting in a statistical significance

Stepwise model: ORs/miR-21 are calculated for 0.001 unit increase; ORs/SOD2 are calculated for 100 unit increase; ORs/ROS are calculated for 0.01 unit increase

## Discussion

In an era in which blood glucose measurements are critical for prediabetes and diabetes diagnoses, we developed alternative methods based on miRNAs to predict the glycaemic status that reflects the ROS damage index. In the present work, a double face for circulating miR-21 has emerged: while a miR-21-based approach detects glycaemic impairments in populations with high risk to develop diabetes, the associations between circulating miR-21 and plasmatic Ox-S-induced damage revealed putative pathogenic mechanisms. Many clinical diagnoses currently involve objective assessment of biochemical variables and do not routinely examine miRNAs; this is possibly due to the lack of standard methods, adequate reference intervals and efficient normalization factors. The current diagnostic criteria in diabetes have been based on ADA and WHO criteria [4] but international consensus for the definition of prediabetes is lacking [30].

Since dysglycaemic conditions may remain undetected for many years while silently promoting disease progression and cardiovascular events, a parallel analysis of miRNA values may be helpful for identifying prediabetes and provide initial clues for dysglycaemic state detection.

miR-21 plasma levels are still under characterized in the diabetic population. The MIR21 gene is located in the 3′-UTR end of the TMEM49 gene on chromosome 17q23.2 [9]. It has been implicated in diabetic retinopathy [31], in cardiovascular disease [32], in atherosclerotic plaques [33] and kidney fibrosis [34], and it has been shown to influence PTEN expression [35] in renal mesangial cells exposed to high glucose, and beta cell apoptosis [36]. In addition, miR-21 exerts its deleterious actions in myocardial ischaemic-reperfused mice [37], diabetic nephropathy [11], and insulin resistance (IR) initiation [11]. In addition, the protective effects of miR-21 silencing fall on neovascularisation and inflammation in diabetic retinopathy, evidenced by “knock-down” experiments [38]. Beside its effects in T2D, miR-21 has deleterious effects on islets of prediabetics and non-obese diabetic (NOD) mice and on beta-cell function in T1D [39]; additionally, in contrast to what occurred in the data profiling of Zampetaki et al. [7] in which was found a reduced level of miR-21, a recent work by Seyhan et al. [40] highlighted increased miR-21 levels in plasma of T1D and T2D subjects, as a reflection of islet inflammation; this discrepancy could be due to different normalisation strategy among laboratory.

In this study, we revealed the associations between circulating miR-21 and glycaemic impairments and provided novel and valuable insights into the molecular characterization of IGT status. First, we found that circulating miR-21 is increased in IGT subjects as an effect of glucose intolerance; this is known because the logistic model adjusted only for ROS demonstrated that miR-21 does not lose statistical significance.

It is worth noting, as demonstrated by our ROC analyses, that plasma expression levels of miR-21 accurately differentiate IGT and T2D subjects from normoglycaemic controls. When coupled with canonical glucose markers, miR-21 plasma levels may represent a useful tool with strong prognostic utility. In addition, our results showed a positive correlation between miR-21 and post-prandial glucose levels (1hPG and 2hPG) with ROS and revealed an association with the insulin resistance index (Table 2). Accordingly, high levels of insulin and glucose are regularly detected in insulin-responsive tissues from T2D individuals [41]. All these findings allow us to speculate that miR21 expression is linked to the deterioration of insulin-resistance within the pathophysiologic progression from normoglycaemic conditions to IGT and T2D. As HbA1c predicts the risk of developing complications, we sought correlations between miR-21 and HbA1c; no correlation was found between these factors, suggesting that the associations are not linked directly to long-term tissue damage. Highlighting these findings, we posit that miR-21 is a premature index of metabolic derangements. Notably, our correlation analysis showed an inverse link between miR-21 and SOD2 levels ( = − 0.3, p = 0.003), corroborating the hypothesis that miR-21 promotes the suppression of antioxidant signalling that normally limits ROS damage [23].

In diabetes, ROS is an important feature of cardiovascular complication onset [42] and has previously been proposed to be an important trigger for insulin resistance, although evidence for a causal role is lacking.

In our results, plasmatic ROS production, quantitatively measured by EPR that provides an absolute quantification of ROS, was increased in IGT and in newly diagnosed T2D, although with higher variability in IGT than T2D, likely because, once the glycaemic damage process becomes more irreversible, the value of miR-21 is limited in defined range; analysis of miR-21 on the large-scale population might unravel this point. As reported in our previous work, miR-21 could be an important modulator of ROS homeostasis and antioxidant pathways, and defective antioxidant responses are one of the major causes of cellular damage [24]. In diabetes, hyperglycemia often inhibits the defensive machinery [43, 44] accompanied by increased lipid peroxidation and reduced endogenous antioxidant levels in diabetic patients compared to controls [45]; it has also been appreciated that antioxidant defences are reduced in diabetics and in healthy controls during 2hPG tests [46], suggesting a role for acute hyperglycaemia in inhibition of the antioxidant systems. The main role of SOD2 is to scavenge superoxide radicals, and it exerts its antioxidant action protecting against cell death and tissue injury. As clearly demonstrated in the logistic model adjusted for age and BMI, increased SOD2 levels lead to reduced risk of developing prediabetes and diabetes (Table 4). We showed that miR-21 interferes with SOD2 expression, affects the antioxidant response systems, and may lead to mitochondrial dysfunction. Since systemic glucose metabolism converges in mitochondria due to insulin signalling required for normal mitochondrial function, it is clear that an impairment of this metabolic function during insulin resistance may be the causal in disease progression [47]. Insulin impairment found in our patients may have caused abnormalities in both mitochondrial biogenesis and function, including SOD2 impairments in detoxifying superoxide anions.

Lipids undergo peroxidation in the presence of ROS. Increased levels of HNE in plasma and biological fluids are observed in many human diseases, including diabetes complications [48] and atherosclerosis [49]. Interestingly, in this work we noticed an increase in plasma lipid peroxidation adducts (HNE) in IGT and newly diagnosed T2D subjects. Moreover, our evidence of reduced levels of SOD2 highlights the issue of inefficient detoxification of lipids by endogenous plasma activities which may provoke damage to critical targets. To strengthen this hypothesis contributes the correlation analysis among HNE and ROS ( = 0.33; p = 0.002), TAG and glycaemic parameters (Table 2). Consistent with our findings, TAG represents the most susceptible lipid class to ROS attack, although their correlation with ROS levels was not significant ( = 0.2, p = 0.05). In this context, we also found a reduced amount of SOD2 in IGT; however, in this group, HNE levels are still stabilized. This evidence suggests that although SOD2 was reduced, less antioxidant system functioning occurred in IGT.

Moreover, SOD2 correlates positively with glycaemic parameters and negatively with circulating miR-21, corroborating the idea of a miR-21-dependent defective mechanism of antioxidant response that may silence antioxidant responses in plasma as well as endothelial cells [23]. In addition, we identified the best model for the association with IGT which resulted from a stepwise logistic regression including miR-21, SOD2 and ROS (Table 5). Even in this model, miR-21 was associated with IGT; specifically, for each 0.001 unit increase of miR-21, we detected a 9% increase in the risk of IGT compared to NGT.

Altogether, our data strongly suggest that miR-21 is an early predictor of ROS damage prior to the onset of diabetes. These elements strongly argue in favour of using circulating miR-21 as a screening tool in prevention initiatives in middle-aged individuals. To the best of our knowledge, this is the first study that has tested the associations between circulating miR-21 and levels of the ROS/SOD2 axis.

A number of methodological issues should be improved to verify that circulating miRNAs can be used as diagnostic criteria for prediabetes and diabetes. However, understanding how multiple events converge to influence glycaemic impairment initiation might be important for uncovering mechanisms underlying diabetes progression. In particular, the interactions between miR-21 and biochemical signals, their regulation and dynamics, including how they drive glucose abnormalities and insulin resistance mechanisms, needs to be deciphered in greater detail.

## Conclusions

In conclusion, this work demonstrated the discriminatory ability of miR-21 in a model of prevalent prediabetes. Accordingly, elevated levels of miR-21 are associated with increased abundance of ROS and reduced SOD2 antioxidant defence. Overall, these findings suggest that an epigenetic approach is a feasible strategy for identifying new biomarkers that juxtapose canonical measures in the early detection of metabolic abnormalities.

## Additional file

**Additional file 1.** Absorbance (Abs) of plasma samples read at 375 nm and 414 nm wavelength using a spectrophotometer [26, 27]. The ratio between absorbance at 414 nm and 375 nm was calculated. The ratio major than 1.4 was considered sample hemolyzed [26]. Further, we read at 541 nm and 576 nm in order to verify the high levels of hemolysis [26, 27].

## Abbreviations

2hPG: 2h plasma glucose
3′-UTR: 3′-untranslated region
ADA: American Diabetes Association
Cel-miR-39: *Caenorhabditis elegans* miR-39
DIAPASON: diabetes prediction and screening observational
EPR: electron paramagnetic resonance
EV: extracellular vesicle
FINDRISC: finnish diabetes risk score
FPG: fasting plasma glucose
HNE: 4-hydroxynonenal
HbA1c: glycated haemoglobin
IGT: impaired glucose tolerance
IR: insulin resistance
microRNA: miRNA
NGT: normoglycemic tolerance
OGTT: oral glucose tolerance test
OR: odd ratio
Ox-S: oxidative stress
ROC: receiving operative characteristic
ROS: reactive oxygen species
SOD2: superoxide dismutase 2
T2D: type 2 diabetes

## References

[1] Karam BS, Chavez-Moreno A, Koh W, Akar JG, Akar FG (2017). Oxidative stress and inflammation as central mediators of atrial fibrillation in obesity and diabetes. Cardiovasc Diabetol.

[2] Giacco F, Brownlee M (2010). Oxidative stress and diabetic complications. Circ Res.

[3] Ali MK, Bullard KM, Saydah S, Imperatore G, Gregg EW (2018). Cardiovascular and renal burdens of prediabetes in the USA: analysis of data from serial cross-sectional surveys, 1988–2014. Lancet Diabetes Endocrinol.

[4] American Diabetes A (2015). (2) Classification and diagnosis of diabetes. Diabetes Care.

[5] Barry E, Roberts S, Oke J, Vijayaraghavan S, Normansell R, Greenhalgh T (2017). Efficacy and effectiveness of screen and treat policies in prevention of type 2 diabetes: systematic review and meta-analysis of screening tests and interventions. BMJ.

[6] American Diabetes A (2017). Standards of medical care in diabetes-2017 abridged for primary care providers. Clin Diabetes.

[7] Zampetaki A, Kiechl S, Drozdov I, Willeit P, Mayr U, Prokopi M, Mayr A, Weger S, Oberhollenzer F, Bonora E (2010). Plasma microRNA profiling reveals loss of endothelial miR-126 and other microRNAs in type 2 diabetes. Circ Res.

[8] Prattichizzo F, Giuliani A, De Nigris V, Pujadas G, Ceka A, La Sala L, Genovese S, Testa R, Procopio AD, Olivieri F (2016). Extracellular microRNAs and endothelial hyperglycaemic memory: a therapeutic opportunity?. Diabetes Obes Metab.

[9] La Sala L, Micheloni S, De Nigris V, Prattichizzo F, Ceriello A (2018). Novel insights into the regulation of miRNA transcriptional control: implications for T2D and related complications. Acta Diabetol.

[10] Bartel DP (2009). MicroRNAs: target recognition and regulatory functions. Cell.

[11] Kato M, Castro NE, Natarajan R (2013). MicroRNAs: potential mediators and biomarkers of diabetic complications. Free Radic Biol Med.

[12] Poy MN, Eliasson L, Krutzfeldt J, Kuwajima S, Ma X, Macdonald PE, Pfeffer S, Tuschl T, Rajewsky N, Rorsman P (2004). A pancreatic islet-specific microRNA regulates insulin secretion. Nature.

[13] Esau C, Davis S, Murray SF, Yu XX, Pandey SK, Pear M, Watts L, Booten SL, Graham M, McKay R (2006). miR-122 regulation of lipid metabolism revealed by in vivo antisense targeting. Cell Metab.

[14] de Candia P, Torri A, Pagani M, Abrignani S (2014). Serum microRNAs as biomarkers of human lymphocyte activation in health and disease. Front Immunol.

[15] Corona-Meraz FI, Vazquez-Del Mercado M, Ortega FJ, Ruiz-Quezada SL, Guzman-Ornelas MO, Navarro-Hernandez RE (2018). Ageing influences the relationship of circulating miR-33a and miR-33b levels with insulin resistance and adiposity. Diab Vasc Dis Res.

[16] Catanzaro G, Besharat ZM, Chiacchiarini M, Abballe L, Sabato C, Vacca A, Borgiani P, Dotta F, Tesauro M, Po A (2018). Circulating microRNAs in elderly type 2 diabetic patients. Int J Endocrinol.

[17] Nunez Lopez YO, Retnakaran R, Zinman B, Pratley RE, Seyhan AA (2019). Predicting and understanding the response to short-term intensive insulin therapy in people with early type 2 diabetes. Mol Metab.

[18] Chien HY, Chen CY, Chiu YH, Lin YC, Li WC (2016). Differential microRNA profiles predict diabetic nephropathy progression in Taiwan. Int J Med Sci.

[19] Spinetti G, Fortunato O, Caporali A, Shantikumar S, Marchetti M, Meloni M, Descamps B, Floris I, Sangalli E, Vono R (2013). MicroRNA-15a and microRNA-16 impair human circulating proangiogenic cell functions and are increased in the proangiogenic cells and serum of patients with critical limb ischemia. Circ Res.

[20] Parrizas M, Brugnara L, Esteban Y, Gonzalez-Franquesa A, Canivell S, Murillo S, Gordillo-Bastidas E, Cusso R, Cadefau JA, Garcia-Roves PM (2015). Circulating miR-192 and miR-193b are markers of prediabetes and are modulated by an exercise intervention. J Clin Endocrinol Metab.

[21] de Candia P, Spinetti G, Specchia C, Sangalli E, La Sala L, Uccellatore A, Lupini S, Genovese S, Matarese G, Ceriello A (2017). A unique plasma microRNA profile defines type 2 diabetes progression. PLoS ONE.

[22] Yan S, Wang T, Huang S, Di Y, Huang Y, Liu X, Luo Z, Han W, An B (2016). Differential expression of microRNAs in plasma of patients with prediabetes and newly diagnosed type 2 diabetes. Acta Diabetol.

[23] La Sala L, Mrakic-Sposta S, Micheloni S, Prattichizzo F, Ceriello A (2018). Glucose-sensing microRNA-21 disrupts ROS homeostasis and impairs antioxidant responses in cellular glucose variability. Cardiovasc Diabetol.

[24] La Sala L, Cattaneo M, De Nigris V, Pujadas G, Testa R, Bonfigli AR, Genovese S, Ceriello A (2016). Oscillating glucose induces microRNA-185 and impairs an efficient antioxidant response in human endothelial cells. Cardiovasc Diabetol.

[25] Franciosi M, De Berardis G, Rossi MC, Sacco M, Belfiglio M, Pellegrini F, Tognoni G, Valentini M, Nicolucci A (2005). Use of the diabetes risk score for opportunistic screening of undiagnosed diabetes and impaired glucose tolerance: the IGLOO (Impaired Glucose Tolerance and Long-Term Outcomes Observational) study. Diabetes Care.

[26] Mensah M, Borzi C, Verri C, Suatoni P, Conte D, Pastorino U, Orazio F, Sozzi G, Boeri M (2017). MicroRNA based liquid biopsy: the experience of the plasma miRNA signature classifier (MSC) for lung cancer screening. J Vis Exp.

[27] Kirschner MB, Edelman JJ, Kao SC, Vallely MP, van Zandwijk N, Reid G (2013). The impact of hemolysis on cell-free microRNA biomarkers. Front Genet.

[28] Mrakic-Sposta S, Gussoni M, Montorsi M, Porcelli S, Vezzoli A (2014). A quantitative method to monitor reactive oxygen species production by electron paramagnetic resonance in physiological and pathological conditions. Oxid Med Cell Longev.

[29] DeLong ER, DeLong DM, Clarke-Pearson DL (1988). Comparing the areas under two or more correlated receiver operating characteristic curves: a nonparametric approach. Biometrics.

[30] Makaroff LE (2017). The need for international consensus on prediabetes. Lancet Diabetes Endocrinol.

[31] Qing S, Yuan S, Yun C, Hui H, Mao P, Wen F, Ding Y, Liu Q (2014). Serum miRNA biomarkers serve as a fingerprint for proliferative diabetic retinopathy. Cell Physiol Biochem.

[32] Thum T, Gross C, Fiedler J, Fischer T, Kissler S, Bussen M, Galuppo P, Just S, Rottbauer W, Frantz S (2008). MicroRNA-21 contributes to myocardial disease by stimulating MAP kinase signalling in fibroblasts. Nature.

[33] Cao J, Zhang K, Zheng J, Dong R (2015). MicroRNA-146a and -21 cooperate to regulate vascular smooth muscle cell proliferation via modulation of the Notch signaling pathway. Molecular medicine reports.

[34] Chau BN, Xin C, Hartner J, Ren S, Castano AP, Linn G, Li J, Tran PT, Kaimal V, Huang X (2012). MicroRNA-21 promotes fibrosis of the kidney by silencing metabolic pathways. Sci Transl Med.

[35] Dey N, Das F, Mariappan MM, Mandal CC, Ghosh-Choudhury N, Kasinath BS, Choudhury GG (2011). MicroRNA-21 orchestrates high glucose-induced signals to TOR complex 1, resulting in renal cell pathology in diabetes. J Biol Chem.

[36] Sims EK, Lakhter AJ, Anderson-Baucum E, Kono T, Tong X, Evans-Molina C (2017). MicroRNA 21 targets BCL2 mRNA to increase apoptosis in rat and human beta cells. Diabetologia.

[37] Roy S, Khanna S, Hussain SR, Biswas S, Azad A, Rink C, Gnyawali S, Shilo S, Nuovo GJ, Sen CK (2009). MicroRNA expression in response to murine myocardial infarction: miR-21 regulates fibroblast metalloprotease-2 via phosphatase and tensin homologue. Cardiovasc Res.

[38] Chen Q, Qiu F, Zhou K, Matlock HG, Takahashi Y, Rajala RVS, Yang Y, Moran E, Ma JX (2017). Pathogenic role of microRNA-21 in diabetic retinopathy through downregulation of PPARalpha. Diabetes.

[39] Roggli E, Gattesco S, Caille D, Briet C, Boitard C, Meda P, Regazzi R (2012). Changes in microRNA expression contribute to pancreatic beta-cell dysfunction in prediabetic NOD mice. Diabetes.

[40] Seyhan AA, Nunez Lopez YO, Xie H, Yi F, Mathews C, Pasarica M, Pratley RE (2016). Pancreas-enriched miRNAs are altered in the circulation of subjects with diabetes: a pilot cross-sectional study. Sci Rep.

[41] Buren J, Liu HX, Lauritz J, Eriksson JW (2003). High glucose and insulin in combination cause insulin receptor substrate-1 and -2 depletion and protein kinase B desensitisation in primary cultured rat adipocytes: possible implications for insulin resistance in type 2 diabetes. Eur J Endocrinol.

[42] Sugamura K, Keaney JF (2011). Reactive oxygen species in cardiovascular disease. Free Radic Biol Med.

[43] Ceriello A, Morocutti A, Mercuri F, Quagliaro L, Moro M, Damante G, Viberti GC (2000). Defective intracellular antioxidant enzyme production in type 1 diabetic patients with nephropathy. Diabetes.

[44] Ceriello A, Esposito K, Piconi L, Ihnat MA, Thorpe JE, Testa R, Boemi M, Giugliano D (2008). Oscillating glucose is more deleterious to endothelial function and oxidative stress than mean glucose in normal and type 2 diabetic patients. Diabetes.

[45] Santini SA, Marra G, Giardina B, Cotroneo P, Mordente A, Martorana GE, Manto A, Ghirlanda G (1997). Defective plasma antioxidant defenses and enhanced susceptibility to lipid peroxidation in uncomplicated IDDM. Diabetes.

[46] Ceriello A, Bortolotti N, Crescentini A, Motz E, Lizzio S, Russo A, Ezsol Z, Tonutti L, Taboga C (1998). Antioxidant defences are reduced during the oral glucose tolerance test in normal and non-insulin-dependent diabetic subjects. Eur J Clin Invest.

[47] Lowell BB, Shulman GI (2005). Mitochondrial dysfunction and type 2 diabetes. Science.

[48] Leiter LA, Ceriello A, Davidson JA, Hanefeld M, Monnier L, Owens DR, Tajima N, Tuomilehto J, International Prandial Glucose Regulation Study G (2005). Postprandial glucose regulation: new data and new implications. Clin Ther.

[49] Leonarduzzi G, Chiarpotto E, Biasi F, Poli G (2005). 4-Hydroxynonenal and cholesterol oxidation products in atherosclerosis. Mol Nutr Food Res.

