# Supplementary material for: Circulating microRNA-21 is an early predictor of ROS-mediated damage in subjects with high risk of developing diabetes and in drug-naïve T2D
Source: Cardiovasc Diabetol. 2019 Feb 25;18:18. doi: 10.1186/s12933-019-0824-2 (PMC6388471; doi:10.1186/s12933-019-0824-2)
Supplement: Supplementary file 1 — Additional file 1. Absorbance (Abs) of plasma samples read at 375 nm and 414 nm wavelength using a spectrophotometer [26, 27]. The ratio between absorbance at 414 nm and 375 nm was calculated. The ratio major than 1.4 was considered sample hemolyzed [26]. Further, we read at 541 nm and 576 nm in order to verify the high levels of hemolysis [26, 27]. [file 12933_2019_824_MOESM1_ESM.pdf]

Supplementary file

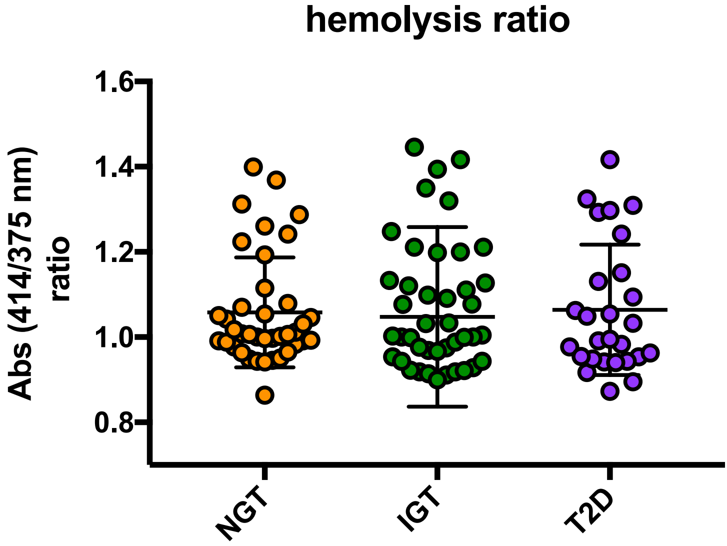

Absorbance (Abs) of plasma samples read at 375 nm and 414 nm wavelength using a spectrophotometer [26, 27]. The ratio between absorbance at 414 nm and 375 nm was calculated. The ratio major than 1.4 was considered sample hemolyzed [26]. Further, we read at 541 nm and 576 nm in order to verify the high levels of hemolysis [26, 27].

Kirshen et al. 2013 [27]
Mensah et al. 2017 [26]
